# Large and small financial incentives may motivate COVID-19 vaccination: A randomized, controlled survey experiment

**DOI:** 10.1371/journal.pone.0282518

**Published:** 2023-03-17

**Authors:** Jessica Fishman, David S. Mandell, Mandy K. Salmon, Molly Candon

**Affiliations:** 1 Perelman School of Medicine, University of Pennsylvania, Philadelphia, Pennsylvania, United States of America; 2 Message Effects Lab, Annenberg School for Communication, University of Pennsylvania, Philadelphia, Pennsylvania, United States of America; 3 Leonard Davis Institute of Health Economics, University of Pennsylvania, Philadelphia, Pennsylvania, United States of America; Northeastern University, UNITED STATES

## Abstract

**Introduction:**

Experts continue to debate how to increase COVID-19 vaccination rates. Some experts advocate for financial incentives. Others argue that financial incentives, especially large ones, will have counterproductive psychological effects, reducing the percent of people who want to vaccinate. Among a racially and ethnically diverse U.S. sample of lower income adults, for whom vaccine uptake has lagged compared with higher income adults, we empirically examine such claims about relatively large and small guaranteed cash payments.

**Methods:**

In 2021, we conducted a randomized, controlled experiment among U.S. residents with incomes below $80,000 who reported being unvaccinated against COVID-19. Study participants were randomized to one of four study arms. In two arms, respondents first learned about a policy proposal to pay $1,000 or $200 to those who received COVID-19 vaccination and were then asked if, given that policy, they would want to vaccinate. In the two other arms, respondents received either an educational message about this vaccine or received no vaccine information and were then asked if they wanted to vaccinate for COVID-19. The primary analyses estimated and compared the overall percentage in each study arm that reported wanting to vaccinate for COVID-19. In other analyses, we estimated and compared these percentages for subgroups of interest, including gender, race/ethnicity, and education.

**Main results:**

Among 2,290 unvaccinated adults, 79.7% (95%CI, 76.4–83.0%) of those who learned about the proposed $1,000 payment wanted to get vaccinated, compared with 58.9% (95%CI, 54.8–63.0%) in the control condition without vaccine information, a difference of 20 percentage points. Among those who learned of the proposed $200 payment, 74.8% (95% CI, 71.3–78.4%) wanted to vaccinate. Among those who learned only about the safety and efficacy of COVID-19 vaccines, 68.9% (95% CI, 65.1–72.7%) wanted to vaccinate. Findings were consistent across various subgroups.

**Discussion:**

Despite several study limitations, the results do not support concerns that the financial incentive policies aimed to increase COVID-19 vaccination would have counterproductive effects. Instead, those who learned about a policy with a large or small financial incentive were more likely than those in the control condition to report that they would want to vaccinate. The positive effects extended to subgroups that have been less likely to vaccinate, including younger adults, those with less education, and racial and ethnic minorities. Financial incentives of $1,000 performed similarly to those offering only $200.

## Introduction

During the SARS-CoV-2 pandemic, proposals to pay people for vaccinating [1–3] have inspired heated debate [4–6]. Critics argue that these financial incentives, especially those offering large payments, would reduce the percent of the U.S. population wanting to vaccinate against COVID-19. Critics have also warned that such policies would be especially counterproductive among groups that have been least likely to vaccinate, including lower income groups and Black and Latinx Americans [4–6].

When explaining why they believe financial incentives would decrease the number of Americans who would want to vaccinate, experts have offered various reasons. For example, some experts contend that financial incentives are likely to give the impression that the vaccine lacks inherent value [4–6]. Critics also assert that financial incentives would signal risk and erroneously suggest that the vaccine is not safe and effective, creating suspicions that the government is “bribing” people to vaccinate [4–6]. Similarly, based on a classic psychological theory, some have argued that individuals will judge the incentive terms unappealing because compliance would make them feel they are mere “pawns” to others’ attempts to control their behavior [7, 8].

On the other hand, financial incentives could increase COVID-19 vaccination rates by strengthening vaccine motivation. Financial incentives have had mixed effects in studies designed to increase motivation for various health-related behaviors, such as attending medical appointments, exercising, and donating blood; some of these studies have found financial incentives to be effective whereas others found them to be ineffective or even counterproductive [9–11]. This larger literature begs the question of when and why financial incentives have a positive or negative psychological effect—or any effect at all.

Because critics have argued that policies offering relatively large payment amounts are especially likely to decrease interest in vaccination [4–6], we tested financial incentive policy proposals that offered to pay a relatively large or small payments for COVID-19 vaccination. The larger payment was $1,000, which is part of a policy proposal originally developed by the economist Robert Litan that continues to be debated [1]. Our experiment also tested a $200 payment policy, which was selected because it is similar in size to those offered as compensation for other medical procedures, such as bone marrow donation [9–11].

Studies in the U.S. have begun to examine the effects of financial incentive policies on COVID-19 vaccine motivation, but they have lacked comparisons between relatively small and large payment amounts. (For an exception, see Fishman et al., 2022, which focuses on a different study population [12].) Some studies have exclusively tested the effects of policies offering relatively large payments. For example, U.S. residents were told of a hypothetical policy paying $1,000-$2,000 for COVID-19 vaccination [13]. The effects were measured very early in the pandemic, when a vaccine was not yet available and had unknown safety and efficacy. The current study was conducted when a proven safe and effective vaccine was available. Other research has exclusively tested relatively small payments; in Sweden, for example, incentives equivalent to $25 USD increased vaccination rates [14]. Data from other countries may have limited generalizability to the U.S.

Our research also contributes to the literature by focusing on the malleable, psychological mechanism that predicts future behavior, including COVID-19 vaccination [14–18]. Since vaccination is voluntary and subject to consent, this psychological effect is a rate-limiting; vaccination does not otherwise occur. Not only does our study outcome predict vaccination, but there is no known alternative that has stronger predictive validity [14–18]. As demonstrated by empirically tested causal model pathways, this study outcome is influenced by vaccine attitudes and other cognitions (i.e., perceived norms and self-efficacy towards vaccination). In other words, the study outcome comprehensively and efficiently reflects these underlying cognitions [16, 19].

Furthermore, when the study outcome is limited to vaccination behavior, the effects on the psychological mechanism can be obscured [15–19]. In particular, if vaccination rates do not rise within a study’s follow up period, we cannot infer the strength or direction of psychological effects, which could have been positive, negative or neutral. One might be tempted to conclude the incentives had no positive psychological effect [19], but it would be wrong to do so; incentives could have a positive effect on vaccine motivation, but individuals may not have the ability to overcome the logistical obstacles that can make it difficult to vaccinate in a timely fashion [20–24].

Critics have warned that financial incentives are most likely to decrease interest in vaccination among those populations that are already hesitant [4–6]. In turn, the current study samples lower income individuals, who have been less likely to vaccinate against COVID-19. This lower income subpopulation is also important to study because, compared to those with higher income, they are more likely to experience COVID-19 morbidity and mortality [25]. In addition, it is feasible to implement COVID-19 vaccine incentives for lower income individuals. In fact, some U.S. cities have recently adopted such policies [26].

## Methods

This research was reviewed by the Institutional Review Board at the University of Pennsylvania, which deemed it exempt and waived documentation of participants’ consent. The study design used a randomized, controlled experiment to assign each participant to one of four conditions. Although the primary goal was to compare responses from each study arm, we also consider responses within subgroups, which could generate hypotheses that can be tested in future studies. The experiment was designed to examine if, compared to a control condition, a relatively large incentive, small financial incentive, or a message about vaccine safety and efficacy (which is a typical educational approach to interventions) was associated with an increase or decrease in the percent reporting that they would want to vaccinate against COVID-19.

Study enrollment was conducted through Prolific (www.prolific.co), an online research platform that helps scientists efficiently recruit study participants. Using “crowdsourcing” techniques [27–30], Prolific constantly replenishes and diversifies the participant pool. Participation is arranged on a casual, non-committed basis where individuals can choose to opt-out at any time. Platforms like Prolific have become a dominant data collection technique for psychological and economic experiments [27–31]. Compared to traditional, lab-based experiments, online experiments can produce results that have similar estimates of validity and reliability [32–35]. The effects from classic (and sometimes logistically complex) experiments in psychology and economics have been successfully replicated online [32–34].

With any experiment, data quality can be reduced if an individual participates more times than researchers intended. We relied on automated procedures that make it difficult for the same individual to participate in the experiment more than once [35]. Online experiments have the advantage of automating and standardizing all experimental procedures [31–37]. This automation offers considerable control over how the experiment is conducted, eliminating the chance that questions are asked in slightly different ways that can influence results [36, 37]. In addition, online experiments can reduce enrollment bias because lab-based experiments incur burdensome logistics for many potential respondents that can lead to lower participation rates.

We recruited a theory-based sample (rather than using probability-based sampling) based on income and race/ethnicity. We did not enroll a nationally representative sample for two reasons. First, vaccine uptake is not evenly distributed across the U.S. population [38] and we designed the study to enroll groups whose members have been more likely to be unvaccinated. Second, critics of financial incentives policies have argued that racial and ethnic minorities and lower income groups are specifically at risk for potential negative psychological effects [4–6].

Using Prolific’s automated features, we screened potential participants by their self-reported race/ethnicity and household income. We enrolled those with household incomes less than $80,000—a group that accounts for more than 50% of the U.S. population and that has lower rates of vaccination compared to wealthier Americans [38]. In addition, we recruited similar sample sizes of those who identified as White and not Latinx, Black/African American and not Latinx, and Latinx. We also recruited similar sample sizes based on gender.

When this experiment was conducted in 2021, the FDA had announced emergency authorization of the vaccines, but vaccines were not widely available [38]. To reduce enrollment bias, the focus of the research was not revealed prior to participation in the experiment; individuals did not know *a priori* that questions would concern COVID-19 or vaccines. Instead, when deciding whether to participate, the study was only described as an opportunity to participate in “research.” Respondents were blind to the randomization process, as well as the enrollment criteria.

Using an algorithm that applied a 1:1:1:1 allocation ratio, we randomly assigned unvaccinated individuals to one of four groups. In one of the four groups, participants were notified about a potential government policy to pay $1,000 for COVID-19 vaccination. In a second group, the payment was listed as $200. In the third group, participants received information regarding COVID-19 vaccines’ safety and efficacy (i.e., without any payment policy information). A fourth group served as a control condition and received a general message, with no reference to COVID-19 or vaccination. To control respondent burden, the questionnaire text for each arm was similar in length.

Following exposure to the vaccine policy message, respondents were asked about their interest in vaccination. For instance, as shown in the Supplement, among those assigned to the $200 financial incentive policy, we asked: *“If you were paid $200 to get a COVID-19 vaccine*, *would you want to get vaccinated today*?*”* The response options included “yes” or “no.” This study outcome, which has a dichotomous response option set (and not a third option indicating indecision) is often used in COVID-19 research because, when offered the actual vaccine, one would either accept or decline it and then the patient either receives the injection or not [14, 39, 40].

Respondents also reported standard demographic variables, such as their age, race, ethnicity, and gender. Socio-economic status was reflected in a measure of educational attainment and their personal financial circumstances. The latter is a subjective measure that is often equally or more relevant to psychological outcomes than an objective measure of income [41–43]. The subjective financial circumstances were assessed through two questions: (1) Compared to other Americans, do you think your income is above average, average, or below average; and (2) "When you think of your current financial situation, how worried/stressed do you feel?" [41–43]. We dichotomized the first question by whether respondents reported their income as below average versus average or above average. As noted above, the study’s inclusion criteria relied on an objective indicator of household income.

We collected all data on an external website using the web-based software, Qualtrics. Using STATA 17.0 statistical software, for each of the experiment’s four conditions, we calculated the proportion of respondents who reported that they wanted to vaccinate. This proportion was reported overall, for the primary analysis (as noted in preregistration) and by subgroups for exploratory analyses. The subgroups were specified by age, gender, race/ethnicity, education (college graduate vs. non-graduate), and financial circumstances.

Overall and within subgroups, we assessed whether differences in the proportion wanting to vaccinate across the four study arms were statistically different using group ANOVAs at the 95% level of significance. To allow for pairwise comparison, we also estimated Tukey honestly significant differences (HSD) tests overall and within subgroups; results are available in the Supplement. Tukey HSD tests can assess multiple pairwise differences for the probability of a Type 1 error [44]. Finally, we plotted the proportion wanting to vaccinate and 95% confidence intervals across the four study arms by race/ethnicity and gender; differences were deemed statistically significant if the confidence intervals did not overlap.

## Results

The primary experiment included 2,290 unvaccinated adults, with ages ranging from 18 to 77 years. As displayed in Table 1, the four study arms were well-balanced in demographic and socioeconomic characteristics. In addition, the three racial/ethnic groups—Latinx; Black/African American (non-Latinx) and white (non-Latinx)—had similar socioeconomic characteristics, as shown in the S1 Table.

**Table 1:** Characteristics of the study sample, overall and by study arm.

	Overall (n = 2,290)	Study arm 1 (Control condition): Received no information about payment policy or vaccines (n = 567)	Study arm 2: Received information about a $1,000 payment policy for COVID-19 vaccines (n = 572)	Study arm 3: Received information about a $200 payment policy for COVID-19 vaccines (n = 572)	Study arm 4: Received information about the safety and efficacy of COVID-19 vaccines (n = 579)
Race/ethnicity					
Black (non-Latinx)	33.4%	32.8%	32.9%	34.1%	33.7%
Latinx (Latino/a or Hispanic)	32.6%	32.5%	33.9%	32.3%	31.8%
White (non-Latinx)	34.0%	34.7%	33.2%	33.6%	34.5%
Age: mean (range)	33.1	33.5	32.0	34.3	32.8
Gender					
Male	50.0%	53.3%	51.1%	48.8%	46.8%
Female	50.0%	46.7%	49.0%	51.2%	53.2%
College graduate	52.4%	51.2%	49.3%	51.9%	57.2%
Income less than average	49.7%	47.6%	53.9%	50.0%	47.3%
Financial stress	28.7%	25.2%	32.2%	31.5%	25.9%

As reported in Table 2, 79.7% (95%CI, 76.4–83.0%) of respondents wanted to get vaccinated in the $1,000 payment group and 74.8% (95% CI, 71.3–78.4%) of respondents wanted to get vaccinated in the $200 payment group. By comparison, 68.9% (95% CI, 65.1–72.7%) of respondents who received information regarding safety and efficacy wanted to get vaccinated, and 58.9% (95%CI, 54.8–63.0%) in the control condition wanted to get vaccinated. Group ANOVAs showed that group differences were statistically significant at the 95% level of significance.

**Table 2:** The frequency and percentage of those who want to vaccinate in each condition.

	Control condition	Experimental conditions			Group ANOVA
	No information about vaccination policy or COVID-19 vaccines; % (n) wanting to vaccinate	Information on a $1,000 payment policy for COVID-19 vaccines; % (n) wanting to vaccinate	Information on a $200 payment policy for COVID-19 vaccines; % (n) wanting to vaccinate	Information about the safety and efficacy of COVID-19 vaccines; % (n) wanting to vaccinate	p-value
All respondents	58.9%	79.7%	74.8%	68.9%	<0.001
	(n = 334)	(n = 456)	(n = 428)	(n = 399)	
Race/ethnicity					
Black (non- Latinx)	40.9%	67.0%	63.6%	58.0%	<0.001
Latinx (Latino/a or Hispanic)	68.5%	88.7%	82.2%	80.4%	<0.001
White (non-Latinx)	67.0%	83.2%	79.2%	69.0%	<0.001
Age (years)					
< = 33	64.3%	81.2%	77.7%	69.2%	<0.001
>33	50.7%	76.8%	70.6%	68.3%	<0.001
Gender					
Male	62.6%	84.3%	82.1%	74.2%	<0.001
Female	54.7%	75.0%	67.9%	64.3%	<0.001
Education					
College grad	68.3%	80.9%	77.1%	73.4%	0.004
No college degree	49.1%	78.6%	72.4%	62.9%	<0.001
Income					
< than mean	51.1%	80.5%	75.5%	64.2%	<0.001
> = than mean	66.0%	78.8%	74.1%	73.1%	0.007
Financial stress					
Low	59.7%	79.4%	74.0%	68.3%	<0.001
High	56.6%	80.4%	76.7%	70.7%	<0.001

Pairwise comparisons, using the Tukey HSD tests, indicated that the difference between the $1,000 payment group and the control condition, as well as the difference between the $200 payment group and the control condition, were statistically significant (S2 Table). While we defined the control condition as no receipt of information regarding vaccination policies or COVID-19 vaccines, we also compared the effects of educational information (regarding the safety and efficacy of COVID-19 vaccines) and information regarding payment policies. We note that pairwise differences between the $1,000 payment group and the educational group were statistically significant; differences between the $200 payment group and the safety and efficacy group were also statistically significant.

**Fig 1:**
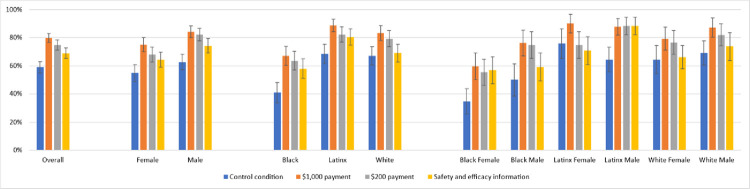
The percentage wanting the COVID-19 vaccine by race/ethnicity and gender.

In each racial/ethnic group, a financial incentive increased the proportion of participants who wanted to vaccinate, compared with the control condition (Fig 1). Among Black participants, 67.0% (95%CI, 60.2–73.8%) in the $1,000 incentive condition and 64.1% (95%CI, 57.3–70.9%) in the $200 incentive condition reported they would get vaccinated, versus 40.9% (95%CI, 33.7–47.9%) of those in the control condition. Among Latinx respondents, 88.7% (95%CI, 84.2–93.2%) reported they would get vaccinated in the $1,000 incentive condition and 82.2% (95%CI, 76.6–87.7%) reported they would get vaccinated in the $200 incentive condition, compared with 68.5% (95%CI, 61.7–75.3%) in the control condition. Among White/non-Latinx participants, 83.2% (95%CI, 77.8–88.5%) reported they would vaccinate for the $1,000 financial incentive, and 79.2% (95%CI, 74.4–85.0%) reported they would for the $200 incentive versus 67.0% (95%CI, 60.4–73.6%) in the control condition.

Table 2 shows that the percent of males wanting to vaccinate was higher than the percent of females. In Fig 1, we plotted the percent of males versus females wanting to vaccinate with each race/ethnicity. Differences in responses were pronounced among Black males compared to Black females, who were the least likely racial/ethnic group to say they wanted to vaccinate. However, none of the differences for males versus females within race/ethnicity were statistically significant.

**Fig 2:**
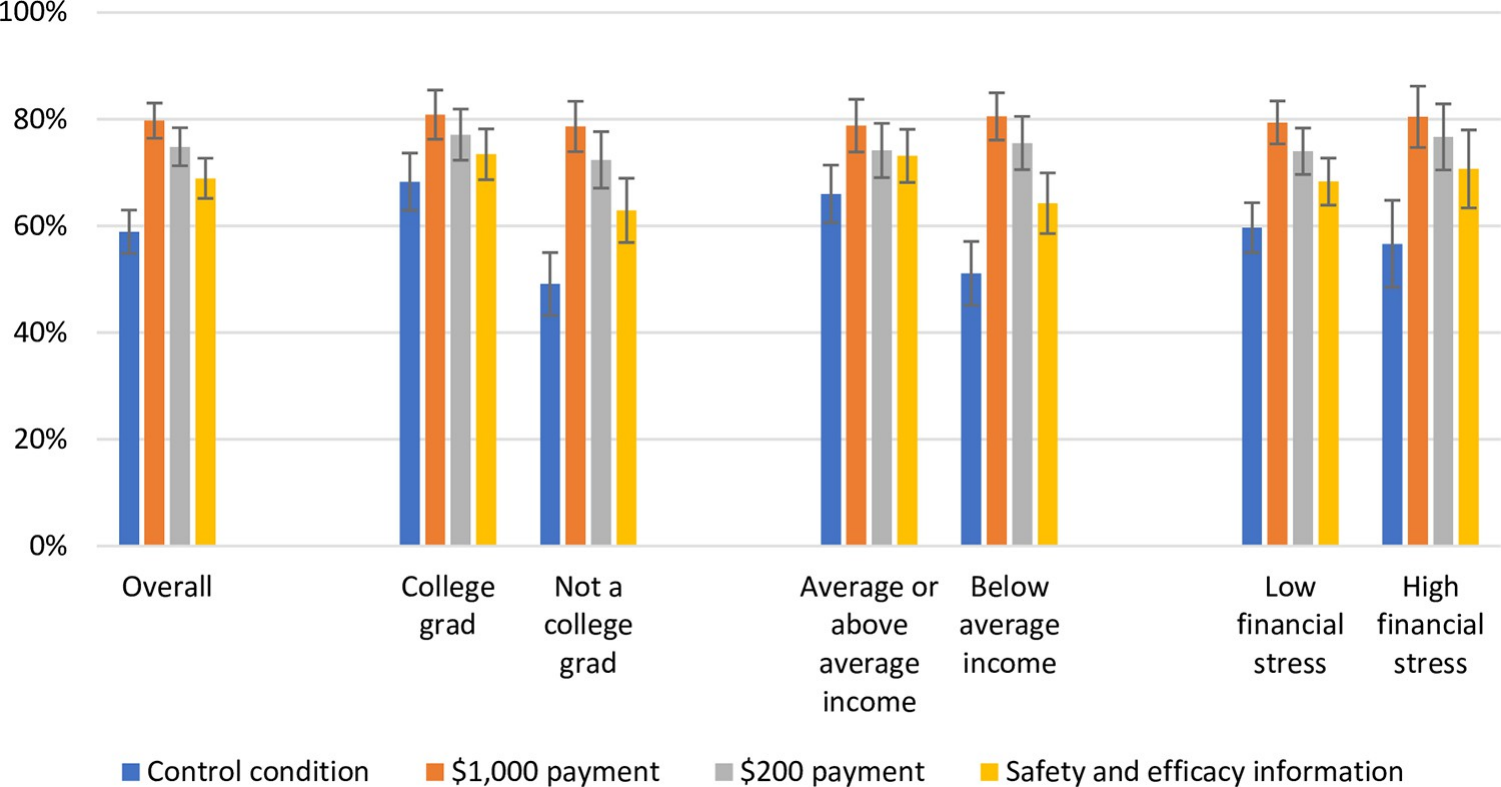
The percentage wanting the COVID-19 vaccine by socioeconomic characteristics.

The total sample included individuals with differing levels of financial hardship, as measured subjectively, even though study enrollment was limited to those with incomes below $80,000. When stratifying by economic and educational characteristics, we found that the percent wanting to vaccinate did not differ appreciably in the three experimental arms (Fig 2). In the control condition, 68.3% (95% CI, 62.9–73.7%) of college graduates wanted to vaccinate compared to 49.1% (95% CI, 43.2–55.0%) of non-college graduates, while 66.0% (95% CI, 60.6–71.4%) of respondents reporting average or above average incomes wanted to vaccinate compared to 51.1% (95% CI, 45.1–57.1%) of respondents reporting below average incomes.

## Discussion

Some experts have warned that financial incentive policies are likely to “backfire” by reducing interest in the COVID-19 vaccine [4–6]. Experts have also argued that this effect would be pronounced among lower income individuals and racial and ethnic minorities [4–6]. However, in this study of such groups, none of the financial incentive policies decreased the percentage who would want to vaccinate for COVID-19. Instead, compared to the control condition, financial incentive policies were associated with a higher percent of respondents wanting to get vaccinated. In the control condition, less than 60% of individuals wanted to vaccinate. With payments, the percentage wanting to vaccinate increased up to 20 percentage points. The positive effects of hypothetical payments held across race/ethnicity, gender, age, and socioeconomic characteristics.

Contrary to the critics’ concerns [4–6], the positive effects were particularly strong among those who identified as Black or Latinx. The effects were also strong among younger adults and those with less education. Our results suggest that there are larger effects for individuals who reported lower income than those reporting higher income. In other research, younger age, less income, education and minority race/ethnicity has been associated with lower rates of COVID-19 vaccination [25, 38].

The effects of the relatively large and small monetary amounts were similar; although the $1,000 payment yielded a slightly stronger effect than the $200 payment, the difference was not statistically significant. While the larger amount was associated with a higher proportion that wanted to vaccinate compared with the smaller amount, the difference may reflect diminishing marginal returns relative to increases in payment size. Various hypotheses could explain this finding and more research is needed to understand why the different payment amounts had similar effects. However, diminishing returns between smaller and larger incentive amounts have also been observed in other vaccination research and in lower income populations [12, 13, 45].

Research on several behaviors, including vaccination, has suggested that educational interventions often fail [46–48]. However, our results suggest that, in this limited circumstance, messages about vaccine safety and efficacy could help. It is not clear why this information had positive effects in this experiment. For example, the effects could be attributed to the specific content and/or unique historical circumstances, such as a sudden global crisis and a novel vaccine. Future research could compare the relative effectiveness of different types of educational information for different types of vaccines.

We can only speculate as to why financial incentives may have a positive psychological effect during the COVID-19 pandemic. Although some unvaccinated Americans are adamantly opposed to vaccination, many who are unvaccinated may be better described as apathetic or ambivalent [49]. In this context, our results suggest that financial incentives could be appealing. Without an attractive incentive, motivation may be lacking.

The financial incentive policies may also decrease the perceived and actual financial costs of vaccination that are disproportionately burdensome for lower income individuals. For example, COVID-19 vaccination sites frequently require appointments that are offered during standard working hours and most employers still do not provide paid time off from work for vaccination [20–22]. In many parts of the country, lower income populations also have few affordable options for transportation to a vaccination site [20–22]. In North Carolina, a quasi-experimental study offered a $25 cash reward, which was designed to offset costs for transportation to a vaccination site [50]. The authors concluded that financial incentive can be a promising strategy to increase vaccination, which was tracked in a pilot program. In our study, the payments for vaccination may be perceived as a subsidy to cover transportation and other logistical costs.

Prisons, which disproportionately incarcerate lower income individuals and individuals from racial and ethnic minorities, have offered financial incentives for COVID-19 vaccination while removing several logistical barriers. For example, several Pennsylvania prisons provided $25 commissary credit to buy clothing, food, or other items [51]. Participating prisons have reported a vaccination rate of over 70%, which is substantially higher than many other U.S. subpopulations, incarcerated or not. Between 2017 and 2019, 26% to 28% of the state’s prisoners would get their flu shots. In October 2020, when prisons provided a $5 incentive, 48.1% of the incarcerated population opted for the flu vaccine [50]. Although caution is warranted when interpreting observational data, these results do not appear to support critics’ concerns that incentives would have unintended negative effects.

### Limitations

Several study limitations should be mentioned. We did not track vaccination status longitudinally, nor did we provide actual payments. Vaccination will depend on how well a financial incentive policy and other relevant vaccine policies are implemented, which was beyond the scope of the present study. For example, with any vaccine policy, success will depend on making individuals adequately aware of the policy [52].

Even if financial incentives increase the proportion of the population that wants to vaccinate, other interventions are needed to help overcome logistical obstacles that can make it difficult for those who want to receive the vaccine in a timely fashion [20–22]. Unpaid time off from work, limited transportation, and other logistical obstacles often make it difficult for lower income populations to vaccinate [20–22]. In these circumstances, financial incentives could increase vaccine motivation but not result in timely vaccination [15, 16]. The current study cannot estimate the moderating effect of logistical obstacles on the relationship between our psychological outcome and actual vaccination. Our study was designed with a psychological variable as the study outcome because the goal was to study this mechanism of vaccine behavior; future experiments would ideally include vaccine intention and vaccination as study outcomes.

Our study also is limited by the fact that we examined the effects of a hypothetical policy. We acknowledge that whether a person receives a vaccine depends not only on whether someone wants to vaccinate (i.e., the current study outcome) but also how difficult it is to obtain vaccination during a certain period [15–17]. When such obstacles are reduced, vaccine motivation has predicted vaccine uptake [15, 17, 18]. Survey research commonly relies on vignettes [53–55], but the psychological effects of an enacted policy could differ. However, hypothetical choices measured in a survey experiment have been validated against real-world behavior, demonstrating support for the external validity of the estimated causal effects [53–55]. Given the magnitude of the positive effects, it is unlikely that the direction of the effects would be reversed from positive to negative.

Our experiment examined the effects of two guaranteed cash payments. Other payment amounts and types of incentives, such as lotteries, may have different psychological effects [12]. In addition, because this study was purposively limited to groups with relatively low COVID-19 vaccination rates, the results may not be generalizable to other domestic or international populations. For example, because money has different connotations and uses in American culture versus other cultures [56], the results may vary between countries. However, a study conducted during Sweden’s and Germany’s pandemic found positive effects of financial incentives on COVID-19 vaccination and vaccination intentions [14, 57]. Trials of incentives for older vaccines have also shown promise [58–60].

Future research could compare the cost-effectiveness of the two policies tested here, along with policies specifying different payment amounts, and estimate the optimal size of a payment. Currently, the cost-benefit ratio can be favorable for even small increases in vaccination from financial incentive programs [61]. Although the larger payments may not be politically feasible, they are within the scope of federally funded financial stimulus payments and if these payments were designed to reward vaccination, it could reduce the cost of any future outlay.

We did not measure political affiliation, which has been associated with COVID-19 vaccination hesitancy. While Republicans are less likely to receive COVID-19 vaccination, partisanship was not a focus for this study because it is not feasible to have vaccine incentives disseminated among those with a particular political affiliation. In contrast, as noted above, vaccine incentive programs have been implemented among lower income populations [26]. We are not aware of vaccine incentive programs that have been implemented specifically for sub-populations defined by race, ethnicity, age or education levels. Although minority race, Latinx, lower age, and less education were associated with positive effects in our study, it is unlikely to be politically feasible (or effective) to implement a policy designed to target one or more of these groups.

## Conclusion

In this randomized, controlled survey experiment, we found that both large and small financial incentives may motivate U.S. adults to vaccinate against COVID-19. This study was designed to examine the psychological mechanism that can help explain whether a financial incentive will succeed or fail, and why. The findings are also relevant to ongoing debates about the relative merits of various policy proposals to increase vaccination rates. Critics of the proposed financial incentive policies for COVID-19 vaccination have warned that such payments, especially large ones, will have counterproductive psychological effects, particularly among racial and ethnic minorities and lower income groups [4–6]. However, our results do not support such concerns. Instead, the results suggest that payments would have positive psychological effects. Financial incentives may therefore be worth considering alongside other equity-promoting strategies.

## Supporting information

S1 Table Sociodemographic characteristics for racial/ethnic subgroups. (DOCX)

S2 Table Tukey test of honestly significant differences (HSD) across study arms, overall and for subgroups. (DOCX)

S1 Data (XLSX)

S2 Data (DOCX)
