# Supplementary material for: Large and small financial incentives may motivate COVID-19 vaccination: A randomized, controlled survey experiment
Source: PLoS One. 2023 Mar 17;18(3):e0282518. doi: 10.1371/journal.pone.0282518 (PMC10022800; doi:10.1371/journal.pone.0282518)
Supplement: S1 Table — (DOCX) [file pone.0282518.s001.docx]

**S1 Table:** **Sociodemographic characteristics for racial/ethnic subgroups**

|  | Black (non-Latinx)  (n=764) | Latinx (Latino/a or Hispanic)  (n=747) | White (non-Latinx)  (n=779) |
| --- | --- | --- | --- |
| Household income <$80,000 | 100% | 100% | 100% |
| Less than mean income level | 50.0% | 44.3% | 54.6% |
| High financial stress | 30.0% | 27.0% | 29.0% |
| Graduated college | 55.0% | 46.3% | 55.7% |
| Male | 43.7% | 58.4% | 48.0% |
| Mean Age (range) | 31.5 | 28.9 | 38.9 |
