# Supplementary material for: Large and small financial incentives may motivate COVID-19 vaccination: A randomized, controlled survey experiment
Source: PLoS One. 2023 Mar 17;18(3):e0282518. doi: 10.1371/journal.pone.0282518 (PMC10022800; doi:10.1371/journal.pone.0282518)
Supplement: S2 Table — (DOCX) [file pone.0282518.s002.docx]

**S2 Table: Tukey test of honestly significant differences (HSD) across study arms, overall and for subgroups**

|  | Study arm 1. No information about vaccination policy or vaccines; | Study arm 2. Information on a $1,000 payment policy for COVID-19 vaccines; | Study arm 3. Information on a $200 payment policy for COVID-19 vaccines; | Study arm 4. Information about the safety and efficacy of COVID-19 vaccines; |
| --- | --- | --- | --- | --- |
| Overall | 58.9% | 79.7% | 74.8% | 68.9% |
| Tukey HSD test | 2, 3, 4 | 1,4 | 1 | 1,2 |
| Race/ethnicity |  |  |  |  |
| Black (non- Latinx) | 40.9% | 67.0% | 63.6% | 57.9% |
| Tukey HSD test | 2, 3, 4 | 1,4 | 1 | 1,2 |
| Latinx (Latino/a or Hispanic) | 68.5% | 88.7% | 82.2% | 80.4% |
| Tukey HSD test | 2, 3, 4 | 1,4 | 1 | 1,2 |
| White (non-Latinx) | 67.0% | 83.2% | 79.2% | 69.0% |
| Tukey HSD test | 2, 3, 4 | 1,4 | 1 | 1,2 |
| Age (years) |  |  |  |  |
| <=33 | 64.3% | 81.2% | 77.7% | 69.2% |
| Tukey HSD test | 2, 3, 4 | 1,4 | 1 | 1,2 |
| >33 | 50.7% | 76.8% | 70.6% | 68.3% |
| Tukey HSD test | 2, 3, 4 | 1,4 | 1 | 1,2 |
| Gender |  |  |  |  |
| Male | 62.6% | 84.2% | 82.1% | 74.2% |
| Tukey HSD test | 2, 3, 4 | 1,4 | 1 | 1,2 |
| Female | 54.7% | 75.0% | 67.9% | 64.3% |
| Tukey HSD test | 2, 3, 4 | 1,4 | 1 | 1,2 |
| Education |  |  |  |  |
| College grad | 68.3% | 80.9% | 77.1% | 73.4% |
| Tukey HSD test | 2, 3, 4 | 1,4 | 1 | 1,2 |
| No college degree | 49.1% | 78.6% | 72.4% | 62.9% |
| Tukey HSD test | 2, 3, 4 | 1,4 | 1 | 1,2 |
| Income |  |  |  |  |
| < than mean | 51.1% | 80.5% | 75.5% | 64.2% |
| Tukey HSD test | 2, 3, 4 | 1,4 | 1 | 1,2 |
| >= than mean | 66.0% | 78.8% | 74.1% | 73.1% |
| Tukey HSD test | 2, 3, 4 | 1,4 | 1 | 1,2 |
| Financial stress |  |  |  |  |
| Low | 59.7% | 79.4% | 74.0% | 68.3% |
| Tukey HSD test | 2, 3, 4 | 1,4 | 1 | 1,2 |
| High | 56.6% | 80.4% | 76.7% | 70.7% |
| Tukey HSD test | 2, 3, 4 | 1,4 | 1 | 1,2 |
|  |  |  |  |  |

**Note.** Tukey HSD test measures whether pairwise demographics are statistically significant. For example, overall and for each study group, the difference between the control condition and each of the experimental arms is statistically significant.
