# Supplementary material for: Large and small financial incentives may motivate COVID-19 vaccination: A randomized, controlled survey experiment
Source: PLoS One. 2023 Mar 17;18(3):e0282518. doi: 10.1371/journal.pone.0282518 (PMC10022800; doi:10.1371/journal.pone.0282518)
Supplement: S2 Data — (DOCX) [file pone.0282518.s004.docx]

**Caption: Study data**

**Supplement: Materials for Each Experimental Arm and the Control Arm**

**[***Material specifically for those randomized to the control condition:]*

What is this survey about?

Many surveys want to learn people’s opinions about health and well-being. For example, some surveys ask about whether people use aspirin or other medications.

Some surveys ask people about their health-related preferences. Preferences can depend on one’s age or where they live. People have different preferences and we know this can be for good reasons. We are interested in learning your preferences.

If you could get the COVID-19 vaccine for free, would you want to get vaccinated today?

- Yes
- No

[*Material specifically for those randomized to the message that only shares information about vaccine safety and efficacy:]*

Is the vaccine safe?

Yes, this vaccine is safe and it works very well. It is about 95% effective, which means that almost everyone who gets vaccinated will be protected and not get sick.

The safety and efficacy have been studied carefully by those who work independently from the federal government, politicians, and companies making the vaccine. You can’t get COVID-19 from the vaccine because the vaccines do not include any live virus.

If you could get the COVID-19 vaccine for free, would you want to get vaccinated today?

- Yes
- No

[*Material specifically for those randomized to the $1000 incentive message:*]

Will people be paid to get the COVID-19 vaccine?

You might be offered $1,000 to get this vaccine. Advisors to the federal government have been studying whether they should pay people $1,000 for getting the COVID-19 vaccine. Those who vaccinate would get a check or direct deposit of cash. Some in government and business are in favor of these payments because it could encourage people to vaccinate and help the economy.

If you were paid $1,000 to get a COVID-19 vaccine, would you want to get vaccinated today?

- Yes
- No

[*Material specifically for those who were randomized to the $200 payment*:]

Will people be paid to get the COVID-19 vaccine?

You might be offered $200 to get this vaccine. Advisors to the federal government have been studying whether they should pay people $200 if they get the vaccine. Those who vaccinate would get a check or direct deposit of cash. Some in government and business are in favor of these payments because it could encourage people to vaccinate and help the economy.

If you were paid $200 to get a COVID-19 vaccine, would you want to get vaccinated today?

- Yes
- No
